# The Mental Differences between Men and Women

**Published:** 1887-12

**Authors:** 


### THE MENTAL DIFFERENCES BETWEEN MEN AND WOMEN.

Our social system hinges so much upon the relative interests, claims, and capacities of men and women, that too much assiduity cannot be employed in endeavoring to mould public opinion upon a question whose issues are so vital and far-reaching. The Mohammedan regards woman as probably soulless. Needless to add, he makes her a slave. The modern movement for promoting the higher education of women would have been impracticable but for the steady growth in the public mind of a conviction that this enlarged culture was not only feasible but an obvious duty. The miserable travesty of education which was formerly thought good enough for young women—the scraps of history and mythology, the music, dancing, and deportment, so long the staple pabulum at "finishing" schools for young ladies—could have been tolerated only by a generation which had accepted without question or examination the low views of female intellectual power prevailing in a former epoch. Hence such questions as those handled so ably by Mr. Romanes, in his paper recently published in the *Nineteenth Century*, are no mere academic discussions, but problems pregnant with conclusions of immense practical importance.

Mr. Romanes cannot be reckoned as a partizan of either side. He sums up, as he himself admits, "with almost brutal frankness," the various intellectual deficiencies of women, real or supposed, but adds that "these elements of weakness are in chief part due to women as a class not having hitherto enjoyed the same educational advantages as men." We are glad of this admission, which has a very vital bearing upon current controversies. The opponents of the higher education of women are singularly unfortunate in their line of argument when they dwell with apparent satisfaction upon the superiority of men in depth of insight, comprehensiveness of view, originality, creative faculty, and judgment. Granted that in these respects women are, as a class, somewhat inferior to men, the legitimate deduction is not that feminine culture is useless or futile, but rather that it should be encouraged by every means in our power in order that women may, as soon as possible, make up the leeway consequent upon centuries of apathy and of neglect of their intellectual claims.

We are somewhat surprised that Mr. Romanes should attach such paramount importance to the fact of the female brain weighing, on the average, about five ounces (one-tenth of the whole) less than the male. We are hardly prepared to admit either of the premises upon which an argument can be founded for regarding this deficiency as a necessary sign of intellectual inferiority. These premises are, first, that size of brain is a fair gauge of mental power, and, secondly, that the difference of size between the brains of men and women is greater than might be expected from the difference between their bodies. As regards the first point, cerebral physiology is still in too nebulous a condition to enable us to say more than that, as a general rule, idiots have small brains and men of distinguished capacity large brains. When we put aside the *monstra*, either of excess or defect, it is by no means clear that the possession of a few ounces of brain matter above the average is a fair gauge of intellectual pre-eminence. Further, the remarkable conclusions of Ferrier and his school have established a surprising closeness of relation between the brain substance and the muscular system, while they have shed very little light upon the *nexus* between cerebral matter and intellect. It is a very fair question for investigation whether the smaller brain of the woman is not in large part due to her comparative muscular inactivity.

As regards the second point, namely, whether the smaller brain of the woman is not simply part and parcel of her smaller body, we are by no means satisfied with Mr. Romanes' rather dogmatic statement that the deficiency of five ounces is "proportionately a greater difference between the male and the female organisms, as a whole." It is a very open question whether the body weight of the average man does not exceed that of the average woman by fully one-tenth.

The progress of female education during the brief period in which it has been intelligently and thoroughly cultivated is surely very striking. Women are everywhere justifying the hopes of their champions, and disappointing the gloomy vaticinations of their opponents. Their university successes have exceeded the most sanguine anticipations, and such a brilliant achievement as that of Miss Ramsey, at Cambridge, this year, is enough to cause grave questionings to believers in the natural and inherent intellectual inferiority of women.

While we thus take exception to some of Mr. Romanes' lines of argument, we are thoroughly with him in his condemnation of the fatuous notion of some feather-brained reformers that there is no such thing as sex in mind; and that, granted an identity of training and environment, men and women would be in all respects mentally alike We cannot give space to discuss this theory, and must rest content with the statement that biology and psychology alike emphatically repudiate it. We doubt whether woman is necessarily and essentially inferior mentally to man; we are quite sure she is mentally dissimilar. The great maternal function alone could not operate without gradually inducing intellectual differences which are perpetuated by sexual transmission. All experience tends to show that woman shines in intuition, man in judgment; that woman is strongest when impelled by emotion, man when impelled by will; that man is creative, woman administrative; that woman is greatest in self-sacrifice, man in conquest and achievement. Be these difference inherent in sex, or the outcome of evolution, their existence can hardly escape any observer who does not start with some preconceived theory. Mr. Romanes concludes with a careful and dispassionate examination of the question whether the higher culture is often physically injurous to women, but this important subject we must reserve for future consideration.—*British Medical Journal.*

